# Green and Facile Synthesis of Highly Stable Gold Nanoparticles via Hyperbranched Polymer In-Situ Reduction and Their Application in Ag^+^ Detection and Separation

**DOI:** 10.3390/polym10010042

**Published:** 2018-01-03

**Authors:** Xunyong Liu, Chenxue Zhu, Li Xu, Yuqing Dai, Yanli Liu, Yi Liu

**Affiliations:** 1 School of Chemistry and Materials Science, Ludong University, Yantai 264025, Shandong Province, China; m13105202785@163.com (C.Z.); ldxuli@126.com (L.X.); daiyuqing1023@163.com (Y.D.); 2 School of Information and Electronic Engineering, Shandong Technology and Business University, Yantai 264005, Shandong Province, China; yanliliu0727@163.com

**Keywords:** thermoresponsive hyperbranched polymer, gold nanoparticles, in-situ synthesis, colorimetric sensor, silver ions

## Abstract

The development of a green and facile strategy for synthesizing high stable gold nanoparticles (AuNPs) is still highly challenging. Additionally, the main problems regarding AuNPs based colorimetric sensors are their poor selectivity and low sensitivity, as well their tendency to aggregate during their synthesis and sensing process. Herein, we present an in-situ reduction strategy to synthesize thermoresponsive hyperbranched polymer (i.e., Hyperbranched polyethylenimine-terminal isobutyramide (HPEI-IBAm)) functionalized AuNPs. The HPEI-IBAm-AuNPs show excellent thermal stability up to 200 °C, high tolerance of a wide range of pH value (3–13), and high salt resistance. HPEI-IBAm acted as the template, the reducing agent, and the stabilizing agent for the preparation of AuNPs. The HPEI-IBAm-AuNPs can be used as colorimetric sensors for the detection of Ag^+^. In the detecting process, HPEI-IBAm serves as a trigger agent to cause an unusual color change from red to brown. This new non-aggregation-based colorimetric sensor showed high stability (maintaining the color lasting without fading), high selectivity, and high sensitivity with an extremely low detection limit of 7.22 nM and a good linear relationship in a wide concentration range of 0–2.0 mM (*R*^2^ = 0.9921). Significantly, based on the thermoresponsive property of the HPEI-IBAm, the AuNPs/Ag composites can be separated after sensing detection, which can avoid secondary pollutions. Therefore, the green preparation and the applications of the unusual colorimetric sensor truly embody the concepts of energy saving, environmental protection, and sustainable development.

## 1. Introduction

The phenomenal optical and chemical properties of gold nanoparticles (AuNPs) have drawn considerable attention for applications in the fields of chemical sensing [1,2], biomedicine [3,4], catalysis [5,6], and so on. In recent years, AuNPs are the most widely studied colorimetric sensors of Ag^+^ detection due to their high extinction coefficients and sensitive surface plasmon resonance (SPR) sensing characteristics in the visible region [7,8,9,10]. Many methods using citrate reduction [11], hydrazine reduction [12], sodium borohydride reduction [13], and solvent extraction reduction [14] have been developed to prepare AuNPs. However, these small-molecule reducing agents are all highly toxic or unsafe. Therefore, the use of green reducing agents can reduce waste and increase the safety of the sample preparation procedure.

As we all know, AuNPs can be used as colorimetric sensors of Ag^+^ detection based on the color changes between red and blue (or purple) in the dispersion and aggregation of AuNPs [8,9,15], respectively. In the sensing system, AuNPs aggregation triggered only by interactions with a particular target is expected, which can greatly improve the specificity and selectivity of the colorimetric sensor. However, AuNPs prepared by traditional small-molecule reduction methods easily aggregate under the conditions of strong acid, solid state, or excess ion concentrations [16,17], which can interfere with the Ag^+^ detection. Therefore, the stability of AuNPs is a major obstacle to practical applications. The design and preparation of AuNPs, which are stable over a broad range of conditions during storage and utilization, are meaningful and very essential.

Due to the limitations and disadvantages of small-molecule reducing agents, the utilization of hyperbranched polymers as multidentate ligands is an effective approach for preparing high stable AuNPs, because hyperbranched polymers have high steric hindrances and a great number of functional groups [18]. Hyperbranched polyethylenimine (HPEI) and its derivatives as typical hyperbranched polymers having a large amount of amino groups, and a specific spheroid-like shape can stabilize the AuNPs [7,10]. Meanwhile, we all know that the amino groups have good reducibility. Therefore, can HPEI derivatives be used as reductants to prepare AuNPs? If achieved, the green preparation of stabilized AuNPs by in-situ reduction of HAuCl_4_ solution using HPEI derivatives as templates, reducing agents, and stabilizers without the additional step of introducing a toxic reducing agent will be significant. Moreover, it has been reported that the functionalized AuNPs can be used as colorimetric sensors to detect Ag^+^ based on the reduction of Ag^+^ by small-molecule reductant such as citrate, ascorbic acid, etc, to form Ag^0^, which was deposited onto the surface of AuNPs with a color change [7,9,10]. Inspired by the above redox-modulated sensing mechanism, we want to use HPEI derivatives that existed in the AuNPs solution to replace these small-molecule reducing agents, which can simplify the detection process and avoid the use of some toxic reducing agents.

Thermoresponsive polymers-AuNPs composites have attracted much interest from both fundamental and applied research due to their sensitive response to external stimuli, such as temperature, pH, or salts [19,20]. Additionally, the composites can be used as colorimetric sensors for detecting the variation of external stimuli based on the thermo sensitive characters of polymers. Recently, we have reported that HPEI with terminal isobutyramide (IBAm) groups (HPEI-IBAm) showed better thermosensitivity than traditional thermoresponsive linear polymers [21]. However, whether and how we can utilize them as separation materials for enriching or separating AuNPs/analyte composites have not been studied systematically. As we all know, some sensor-Ag^+^ assemblies are difficult to be separated from the sensing system, which can cause seriously secondary pollutions. Therefore, we propose a novel colorimetric sensor based on thermoresponsive HPEI-IBAm that can not only detect Ag^+^ but also achieve the separation of sensor/Ag composites, which provides new ideas for the future design and research of environment-friendly multifunctional AuNPs.

Herein, we report that a kind of hyperbranched polymer, i.e., thermoresponsive HPEI-IBAm, can facilely induce the HAuCl_4_ precursor to spontaneously form well-stabilized AuNPs via a thermal process without the additional step of introducing a reducing agents. Compared with the AuNPs prepared by classical small-molecule reduction as colorimetric sensors of Ag^+^, this strategy had several advantages: (1) Thermoresponsive HPEI-IBAm not only serves as a green reductant and excellent stabilizer to prepare AuNPs via in-situ reduction, but it also serves as a trigger agent to induce an obvious color change due to the reduction of Ag^+^ to Ag^0^ deposited onto the surface of AuNPs, as well as serving as a separating agent to enrich and separate sensor/Ag composites; (2) HPEI-IBAm/AuNPs can realize the integration of the preparation, detection, and separation application, which serves the aims of energy saving, environmental protection, and sustainable development, and provides a basis to further develop multifunctional nano-composites; (3) In-situ formation of AuNPs by HPEI-IBAm with a large number of amino groups and high steric hindrances can greatly improve the stability of AuNPs (even under the conditions of high temperature, acid medium, solid state, and excess ion concentrations), which can avoid the interferences from an acid medium, excess ion concentrations, and so on to improve the selectivity of Ag^+^ detection; (4) The color change of AuNPs sensor from red to brown is more sensitive and selective than that from red to purple or blue (the color change from red to brown is unique and can easily be observed by naked eyes), and therefore this colorimetric sensor exhibits high sensitivity and excellent selectivity toward Ag^+^; (5) The thermoresponsive HPEI-IBAm can separate AuNPs/Ag after the detection application by a simple precipitation or centrifugation process, which can avoid secondary pollutions. To the best of our knowledge, this is the first report to prepare AuNPs, detect Ag^+^, and separate AuNPs/analyte composites simultaneously without the additional step of introducing a reductant and without secondary pollutions. This environment-friendly multifunctional colorimetric sensor really embodies energy saving, environment protection, and sustainable development.

## 2. Materials and Methods

### 2.1. Materials

Hyperbranched polyethylenimine (HPEI) with a number-average molecular weight of 10^4^ g/mol was purchased from Aldrich and dried in a vacuum oven at 40 °C for 12 h prior to use. AgNO_3_ and all other chemicals were analytical grade and purchased from Shanghai Sinopharm Chemical Reagent Corporation.

### 2.2. Characterization

^1^H NMR spectra of the HPEI-IBAm were obtained on a Varian INOVA 500MHz spectrometer. Ultraviolet-visible (UV-vis) absorption spectra were performed using a T6 UV/Vis Spectrophotometer (Purkinje General, Beijing, China). Transmission electron microscopy (TEM) analysis was performed on a Philips (Amsterdam, Netherlands) TECNAI G2 F20 operating at 200 kV. The size of the nanoparticles was measured by dynamic light scattering on a Malvern Instruments (Malvern, UK) Zetasizer Nano-ZS90 at 25 °C. The concentration of Ag^+^ was determined by an Inductively Coupled Plasma spectrometer (ICP-9000, Shimadzu, Kyoto, Japan).

### 2.3. Synthesis of Thermoresponsive HPEI-IBAm

The HPEI-IBAm is prepared using a simple amidation reaction between the amino groups of HPEI and isobutyric anhydride [22,23]. Under the atmosphere of nitrogen, 4.0 g isobutyric anhydride (0.025 mol) is added to 15 mL chloroform solution containing 2.0 g HPEI and 2.87 g triethylamine (0.028 mol). After stirring for 3 h at room temperature, the mixture is maintained at 65 °C for 24 h. The residues were obtained by vacuum distillation from reaction liquid, and their purification were carried out by dialysis against methanol using a benzoylated cellulose membrane with a molecular weight cut off 1000 for 3 days.

### 2.4. In-Situ Preparation of HPEI-IBAm Functionalized AuNPs

Five samples 1–5 were prepared as follows: briefly, 9 mL of HAuCl_4_ (0.29 mM) was added into 6 mL of appropriate concentration of HPEI-IBAm aqueous solution (0.5, 1, 3, 9, and 18 mg/L) with initial molar ratio 0.06:1, 0.12:1, 0.36:1, 1.04:1, and 2.08:1 of HPEI-IBAm to gold (corresponding to samples 1–5, respectively). The reaction was carried out in a nitrogen atmosphere under continuous mechanical stirring at 80 °C for 20 min. The mixture was changed from pale yellow to wine red or pink, indicating the formation of HPEI-IBAm functionalized AuNPs.

### 2.5. Preparation of Citrate-Capped AuNPs

For the purpose of comparison, citrate-capped AuNPs were also synthesized according to the previous publications. Briefly, 5.0 mL of HAuCl_4_ solution (5.34 mM) was added to 135 mL of Milli-Q water in a three-necked flask, heated up to reflux. Then, 10 mL of sodium citrate solution (0.9%) was added to the boiling solution while stirring vigorously. After continuous boiling for 15 min, the reaction mixture was cooled to ambient temperature. The concentration of the AuNPs was estimated by Lambert-Beer’s law (the extinction coefficient of ca. 13 nm AuNPs is 2.7×10^8^ M^−1^·cm^−1^ at 520 nm) [10,24].

### 2.6. Stability Testing of HPEI-IBAm Functionalized AuNPs

#### 2.6.1. Thermal Stability Testing

For thermal stability testing, 4.8 mL of HPEI-IBAm functionalized AuNPs solution was dried into powder state in an oven at a given temperature. Three temperature levels of 110, 140, and 200 °C were applied to thermal stability tests. After drying, 4.8 mL of Milli-Q water was added to redisperse the HPEI-IBAm functionalized AuNPs solid powder for the DLS and UV-vis measurements. The previous drying and dissolving process was repeated to test the thermal stability of the HPEI-IBAm functionalized AuNPs. By contrast, the stability of citrate-capped AuNPs was also tested according to the same steps.

#### 2.6.2. pH Stability Testing

For pH stability testing, 13 samples of 4.8 mL of HPEI-IBAm functionalized AuNPs or citrate-capped AuNPs solution were adjusted to different pH (range from 1.0 to 13.0). Then, the stability of HPEI-IBAm functionalized AuNPs and citrate-capped AuNPs solution under different pH can be evaluated by UV-Vis spectrometry, because the aggregation or dispersion of AuNPs is closely associated with the change of UV-Vis characteristic absorption spectrum. The UV-Vis absorption spectra of the AuNPs solution were recorded, respectively, after placing at room temperature for 1 and 7 days.

#### 2.6.3. The Salt Tolerance Testing

10 samples of 4.8 mL of HPEI-IBAm functionalized AuNPs and citrate-capped AuNPs solution with mass concentration of NaCl from 0 to 12.5 g/L were used to evaluate the stability of the AuNPs solution for salt tolerance. The change in solution color and UV-Vis absorption spectra of two AuNPs systems were recorded to compare their difference in the salt tolerance and tolerant stability.

### 2.7. Colorimetric Sensing of Ag^+^

The colorimetric sensing of Ag^+^ was performed as follows. 0.9 mL of HPEI-IBAm solution (6.0 g/L) was added to the mixture of HPEI-IBAm functionalized AuNPs (3.0 mL) and Ag^+^ (0.9 mL) at different concentrations (5.73 to 2 mM). After equilibrating at boiling temperature for 2 min, the color change and absorption spectra of the solution were recorded by a digital camera and a UV-vis spectrophotometer, respectively. All detecting experiments were performed in triplicate.

## 3. Results

### 3.1. Synthesis and Characterization of HPEI-IBAm

**Scheme 1:**
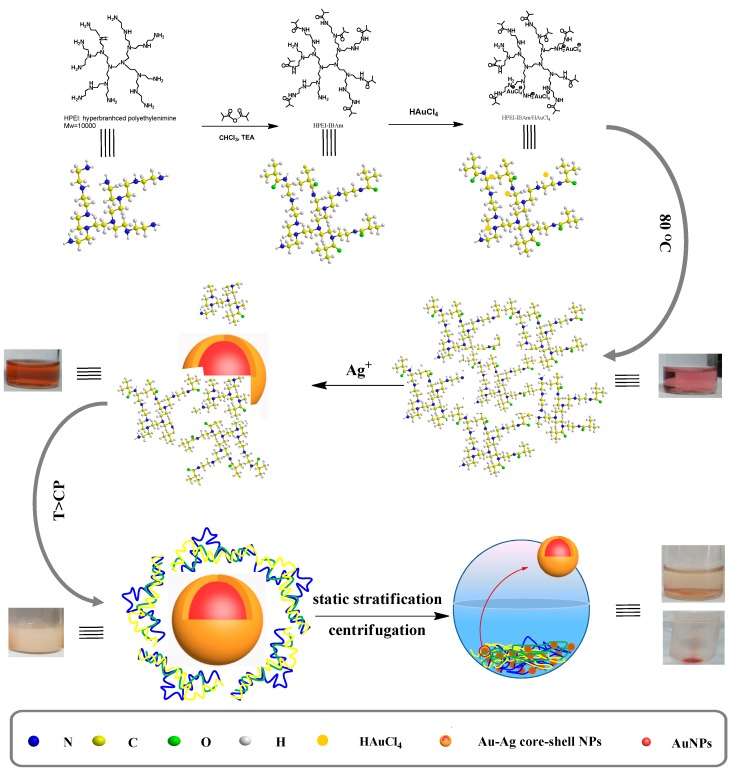
Preparation of thermoresponsive HPEI-IBAm, HPEI-IBAm-AuNPs; the sensing and separating mechanism of HPEI-IBAm-AuNPs toward Ag^+^.

The thermoresponsive HPEI-IBAm was prepared according to the route in Scheme 1. Compared with HPEI, the HPEI-IBAm polymer possessed amide groups that were characterized by ^1^H NMR (Figure S1) and FT-IR (Figure S2). As shown in Figure S1A, HPEI-IBAm exhibits a new signal located at 1.04 ppm that comes from the methyl protons of theisobutyramide groups (a′ in Figure S1A, –NH–CO–CH–(CH_3_)_2_, along with the disappearance of –NH_2_ and NH peaks at 1.4–1.8 ppm (a and b in Figure S1B) of HPEI reactant. Meanwhile, the substitution degree of the amine groups of HPEI is about 83.1% calculated from the integral of the NMR data [22], which indicates that the HPEI-IBAm has not only a large number of isobutyramide groups (giving HPEI-IBAm thermosensitive character) but also many unreacted amine groups (having reducing capacity). The successful synthesis of HPEI-IBAm was further confirmed according to the change of FTIR spectra of HPEI and HPEI-IBAm. In comparison to HPEI, HPEI-IBAm exhibits new bands at around 1633 and 2960 cm^−1^ corresponding to the characteristic C=O stretching vibrations of the –CONH– groups and –CH_3_ stretching vibrations of the –COCH(CH_3_)_2_, respectively. These characterization results indicate that the HPEI-IBAm is successfully prepared by the reaction of HPEI and isobutyric anhydride.

### 3.2. In-Situ Preparation of HPEI-IBAm Functionalized AuNPs

To improve the stability of AuNPs and avoid the limitations and disadvantages of small-molecule reducing agents, the stabilization of AuNPs by polymers as multidentate ligands is one of the most important and effective methods. Over the last few decades, there has been growing interest in using dendritic polymers (especially dendrimers with perfect structure and hyperbranched polymers) as stabilizers or templates to prepare functionalized AuNPs. For example, entrapped dendrimers or stabilized AuNPs can be prepared by the reduction of Au^3+^ inside the dendrimer core [25,26,27,28,29]. However, these AuNPs are very small in size (<5–9 nm in diameter) and difficult to visualize (especially at low concentration) and characterize (since they have no characteristic UV-Vis absorption), and are not applicable to colorimetric detection. The hyperbranched polymer with imperfect structure can prepare large-sized AuNPs due to its relatively weak steric repulsion compared to dendrimers [18].

**Figure 1:**
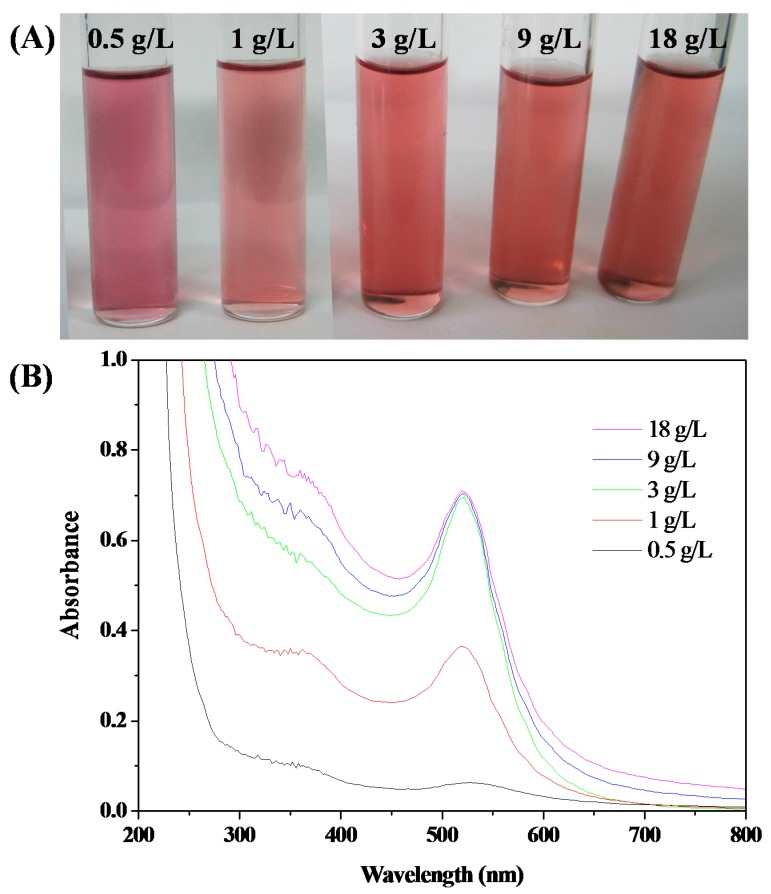
Effect of the concentration of HPEI-IBAm on synthesis of AuNPs: (**A**) the color and (**B**) the UV-Visible spectra.

We used a hyperbranched polymer derivative, i.e., thermoresponsive HPEI-IBAm, as reducing agent to prepare HPEI-IBAm functionalized AuNPs (Scheme 1). In the preparation process, no additional reducing agents were added. The formation of AuNPs depends on the amount of added HPEI-IBAm, because Au^3+^ can be reduced by the unreacted amine groups of HPEI-IBAm. As shown in Figure 1, the color and the maximum UV-vis absorption wavelength of AuNPs solution changed from purple red to red and shifted toward short wavelength (532 to 519 nm), respectively, as the increasing of HPEI-IBAm concentration in the experimental range (0.5 to 18 g/L). When the concentration of HPEI-IBAm was more than 3 g/L, the color and the maximum UV absorption wavelength of AuNPs solution did not significantly change, because all of the Au^3+^ had been reduced completely. Therefore, the concentration of HPEI-IBAm was fixed at 3 g/L (i.e., the initial molar ratio 0.36:1 of HPEI-IBAm to gold) for the preparation of HPEI-IBAm functionalized AuNPs.

**Figure 2:**
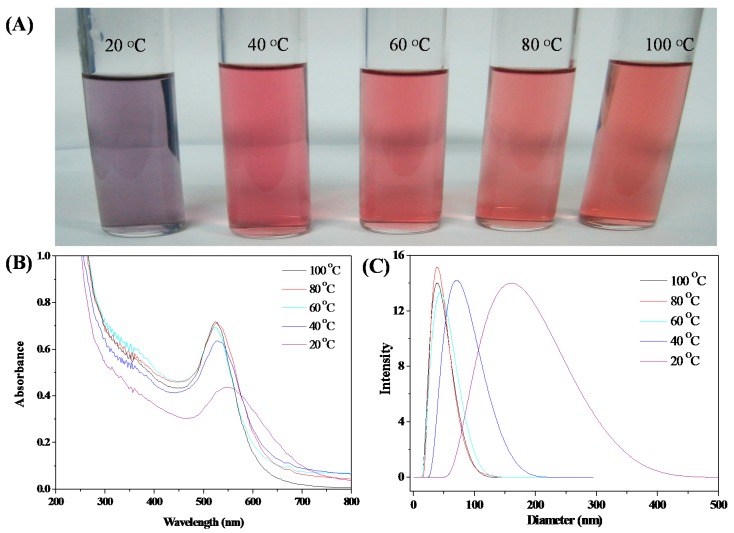
(**A**) Photographs, (**B**) UV-Visible spectra, and (**C**) DLS diagram of HPEI-IBAm functionalized AuNPs when the preparation temperature increased from 20 to 100 °C.

As shown in Figure 2A, when the preparation temperature was set at 20, 40, 60, 80, and 100 °C, respectively, the products were purple (at 20 °C), purple red (at 40 °C) and red (above 60 °C). The AuNPs being purple or purple red are usually unstable and tend to aggregate, resulting in increased particle size and a shift to long wavelength of UV-vis absorption peak. It can be seen from Figure 2B,C that the maximum UV-Vis absorption wavelength and the particle size of AuNPs did not significantly change when the in-situ preparation temperature was higher than 80 °C. Therefore, we select 80 °C to prepare the HPEI-IBAm functionalized AuNPs.

### 3.3. Stability of HPEI-IBAm Functionalized AuNPs

As we all know, noble metal nanoparticles are prone to aggregate due to the influence of Van der Waals force, temperature, pH, and other factors, reducing the stability of NPs, which is also a very challenging problem for all NPs in preparation and application [16]. In our present study, HPEI-IBAm functionalized AuNPs that have a special topology structure, where one gold NP is surrounded with multiple HPEI-IBAm molecules, are prepared by in-situ reaction of HAuCl_4_ in HPEI-IBAm solution to improve their stability.

**Figure 3:**
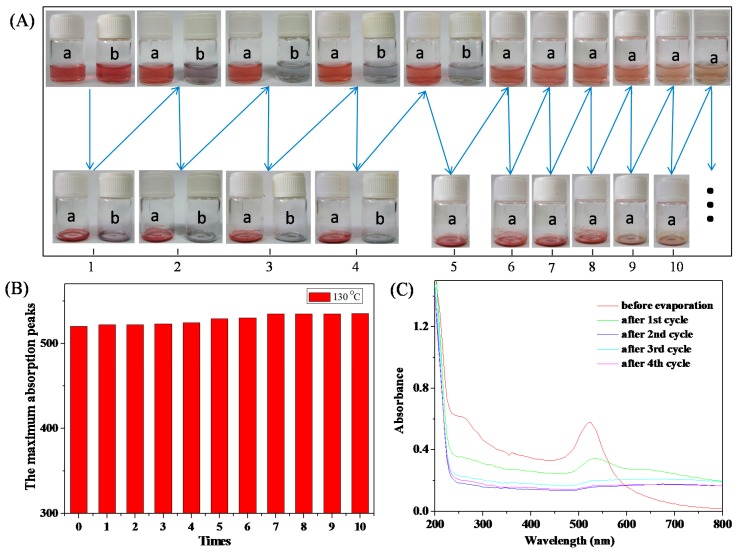
(**A**) Photographs of (a) the HPEI-IBAm functionalized AuNPs and (b) citrate-capped AuNPs after every evaporation at 130 °C and re-dispersion in Milli-Q water; (**B**) the maximum absorption peaks of the HPEI-IBAm functionalized AuNPs after each cycle’s re-dispersion; (**C**) the UV-Vis spectra of the citrate-capped AuNPs after each cycle’s re-dispersion.

As shown in Figure 3 and Figure S3–S6, the HPEI-IBAm functionalized AuNPs showed higher thermal stability than citrate-capped AuNPs. After 10 times of evaporation at 110 or 130 °C and re-dispersion (Figure 3A and Figure S4A), a little red shift (Figure 3B) was shown in the UV-Visible spectra of the HPEI-IBAm functionalized AuNPs. The HPEI-IBAm functionalized AuNPs are stable even at 200 °C after 3 times of drying and re-dispersion as shown in Figure S6. Although the color of the HPEI-IBAm functionalized AuNPs solution changed from red to reddish brown (which was perhaps due to the partial oxidation of the amine groups of HPEI-IBAm) after 4 times of drying and re-dissolution, no aggregation or precipitation was observed. However, the citrate-capped AuNPs showed obvious red shift (Figure 3C) and changed from red to blue (Figure 3A) after 1 time of evaporation at 110–200 °C and re-dispersion, indicating that the citrate-capped AuNPs are extremely unstable when they exist in solid form. Compared with the citrate-capped AuNPs, it can be seen that the HPEI-IBAm by means of its large steric hindrance effect and multi-site protection can significantly improve the thermal stability of AuNPs prepared by in-situ reduction reaction.

**Figure 4:**
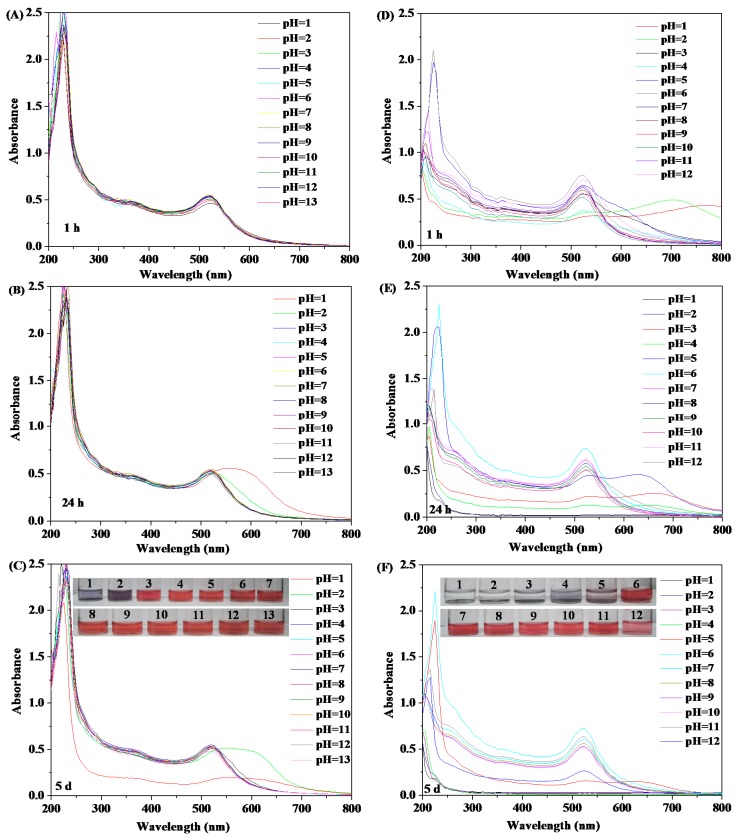
The UV-Vis spectra of the HPEI-IBAm functionalized AuNPs after (**A**) 1 h; (**B**) 24 h and (**C**, Inset shows the color of the HPEI-IBAm functionalized AuNPs) 5 d under different pH value; the UV-Vis spectra of the citrate-capped AuNPs after (**D**) 1 h; (**E**) 24 h; and (**F**, Inset shows the color of the citrate-capped AuNPs) 5 d under different pH value.

It is well known that the pH of the different tested system may vary from acid to alkaline. Therefore, it is extremely important for the probe to remain stable in the widest possible range of pH. To demonstrate their potential applications in sensing, the pH stability of the HPEI-IBAm functionalized AuNPs and the citrate-capped AuNPs was also evaluated here. The HPEI-IBAm functionalized AuNPs can be stable after adjusting the pH 1–13 for 1 h (Figure 4A), and the citrate-capped AuNPs have shown a pronounced red shift after 1 h under the pH 1–2 (Figure 4D). After 24 h, the HPEI-IBAm functionalized AuNPs have a slight red shift only at pH = 1 (Figure 4B). However, the citrate-capped AuNPs have obvious red shift or precipitation at pH range of 1–5 (Figure 4E). After 5 d, the HPEI-IBAm functionalized AuNPs remain stable except for pH 1–2 (Figure 4C), which is mainly because HPEI-IBAm with a large number of amine groups can react with acid. The citrate-capped AuNPs not only precipitated completely at pH range of 1–5, but also decreased their absorption peak intensity at pH = 12 due to the influence of relatively high salt concentration (Figure 4F). Therefore, the HPEI-IBAm functionalized AuNPs have a wider range of acid-base usage than citrate-capped AuNPs, which is more conducive to the practical application of colorimetric detection.

**Figure 5:**
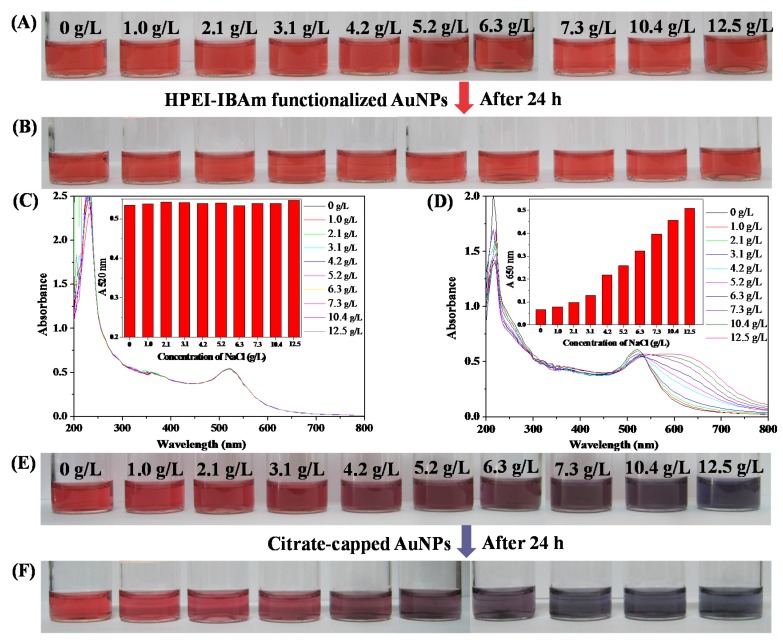
Photographs of the HPEI-IBAm functionalized AuNPs (**A**) before and (**B**) after 24 h under different salt concentrations; the UV-Vis spectra (**C**) of the HPEI-IBAm functionalized AuNPs after 24 h under different salt concentrations (Inset shows the *A*_520_ value of the absorbance at 520 nm versus the concentration of NaCl); the UV-Vis spectra (**D**) of the citrate-capped AuNPs under different salt concentrations (inset shows the *A*_650_ value of the absorbance at 650 nm versus the concentration of NaCl); photographs of the citrate-capped AuNPs (**E**) before and (**F**) after 24 h under different salt concentrations.

Besides the influence of temperature and pH on the stability of AuNPs, the salinity is also an important factor affecting the stability of AuNPs. If the effect of salt causes the aggregation and the color change of AuNPs, it will interfere with their colorimetric detection. Therefore, the AuNPs used for colorimetric detection require good salt tolerance. The salt tolerance of the HPEI-IBAm functionalized AuNPs, and the citrate-capped AuNPs was systematically studied. Experimental results demonstrate that no obvious changes in color (Figure 5A,B) and UV-Vis spectra (Figure 5C and Figure S7) before and after 24 h under different salt concentrations are observed for HPEI-IBAm functionalized AuNPs, indicating that the HPEI-IBAm functionalized AuNPs are very stable against the variation of salinity. However, the citrate-capped AuNPs have obvious red shift (Figure 5D) in UV-Vis spectra, and the A_650_ values of the absorbance at 650 nm increase from 0.067 to 0.509 (Inset Figure of Figure 5D) with the increase of salt concentration, indicating that the citrate-capped AuNPs have aggregated and are unstable to salt. Meanwhile, the color of the citrate-capped AuNPs solution also changed from red to purple or blue (Figure 5E) and became lighter after 24 h (Figure 5F) due to the aggregation and precipitation of AuNPs with the decrease of the A_650_ values (Figure S8) relative to Figure 5D. The contrastive study proves that the HPEI-IBAm plays an important role in stabilizing the AuNPs.

The above experiments show that the HPEI-IBAm functionalized AuNPs prepared by in-situ reaction have higher stability to temperature, pH, and salinity, which would greatly broaden their fields of application.

### 3.4. Colorimetric Sensing of Ag^+^

**Figure 6:**
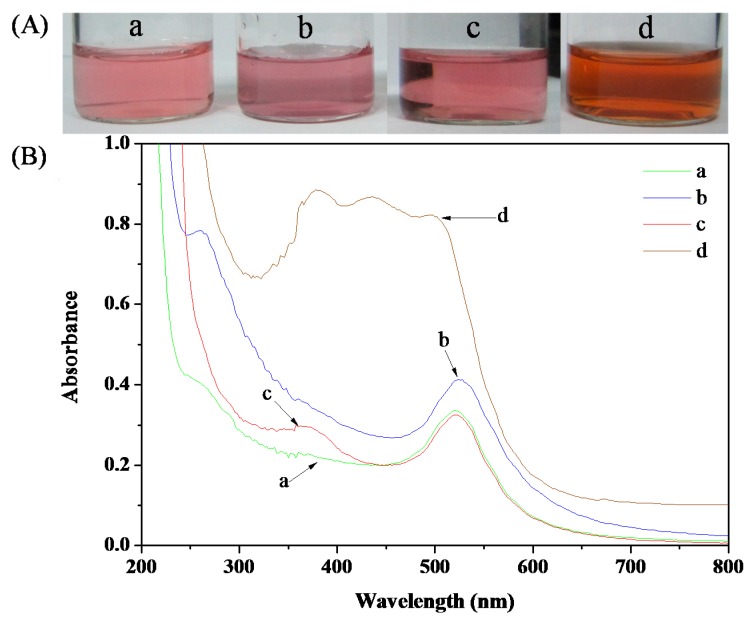
Colorimetric response of the sensor: (**A**) color changes and (**B**) absorption spectra of (a) 1.2 nM citrate-capped AuNPs; (b) 1.2 nM citrate-capped AuNPs with 0.573 mM Ag^+^; (c) 1.2 nM HPEI-IBAm functionalized AuNPs; (d) 1.2 nM HPEI-IBAm functionalized AuNPswith 0.573 mM Ag^+^.

The mechanisms of the conventional AuNPs-based colorimetric sensors for the detection of Ag^+^ are mostly based on the aggregation of AuNPs with a red-to-blue or red-to-purple color change [9,15,30,31]. In our experiments, the citrate-capped AuNPs have slight (Figure 6B-b) and obvious (Figure S9B-c) red shift in UV-Vis spectra with a red (Figure 6A-a) to purple (Figure 6A-b) to blue (Figure S9A-c) color change after the addition of Ag^+^ for 1 and 24 h, respectively. However, many factors, such as strong acid environment, high temperature, high salt, and so on, can also induce the aggregation of AuNPs with a red to blue (or purple) color change, which will interfere with the detection of Ag^+^ and reduce the selectivity of colorimetric sensor. Therefore, developing a colorimetric sensor for Ag^+^ detection based on the non-aggregation of AuNPs is of great significance. It has been reported that the citrate-capped AuNPs can be used as a colorimetric sensor for Ag^+^ detection based on the reduction of Ag^+^ by small-molecule reducing agents such as citrate, ascorbic acid, etc, to form Ag^0^ deposited onto the surface of AuNPs [8,9,10]. Inspired by the redox-modulated sensing mechanism, we use HPEI-IBAm to replace these small-molecule reducing agents, which can avoid using some toxic reductants and stabilize the AuNPs/analyte composites to maintain the color lasting without fading. In this work, after the addition of HPEI-IBAm, the color of the HPEI-IBAm functionalized AuNPs solution changes from red (Figure 6A-c) to brown (Figure 6A-d) in the presence of Ag^+^, which can realize the colorimetric detection of Ag^+^. The brown color is similar to the color of the Au-Ag core-shell NPs (with Au as the core and Ag as the shell). Therefore, the sensing mechanism of this colorimetric sensor can be explained as follows: the reduced Ag^0^ is directly deposited onto the AuNPs surface, forming the Au-Ag core-shell NPs because of the high affinity between Au and Ag and leading to a red to brown color change; meanwhile, the HPEI-IBAm with a high steric hindrance effect can protect Au-Ag core-shell NPs against aggregation (Scheme 1).

**Figure 7:**
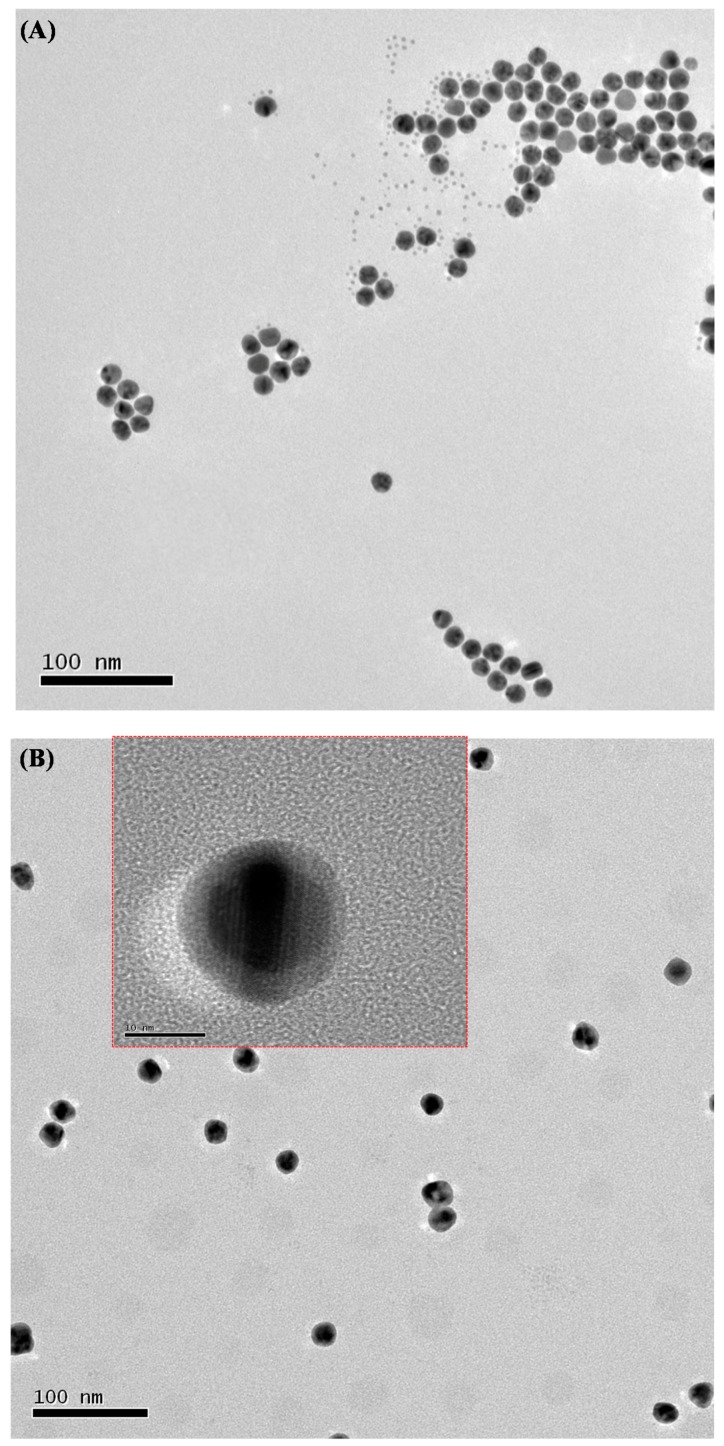
TEM images of (**A**) HPEI-IBAm functionalized AuNPs and (**B**) Au-Ag core-shell NPs (Inset shows a typical HR-TEM image of Au-Ag core-shell NP).

From Curve c in Figure 6B, we can see that the surface plasmon resonance peak of the HPEI-IBAm functionalized AuNPs appears at about 520 nm. The UV-vis spectra of the Au-Ag core-shell NPs (Curve d in Figure 6B) showed a wide plasmon resonance bands, which was consistent with the other previous core-shell NPs. Therefore, from the change of the UV-vis absorption spectra, we could reasonably infer that Ag^0^ was deposited on the preformed Au core surface, forming the Au-Ag core-shell NPs. These changes were further confirmed by the TEM images. It can be seen clearly from the TEM images (Figure 7B) that most of the Au-Ag NPs had a darker central part (Au core) and a clearer outer part (Ag shell). Moreover, the HPEI-IBAm stabilized AuNPs and Au-Ag core-shell NPs shown in Figure 7 were all close to uniform dispersion, and no large aggregation was observed. Therefore, the new non-aggregation-based colorimetric sensor with a unique red-to-brown color change showed higher stability and anti-interference ability than that based on the aggregation of AuNPs with a red-to-blue (or purple) color change.

**Figure 8:**
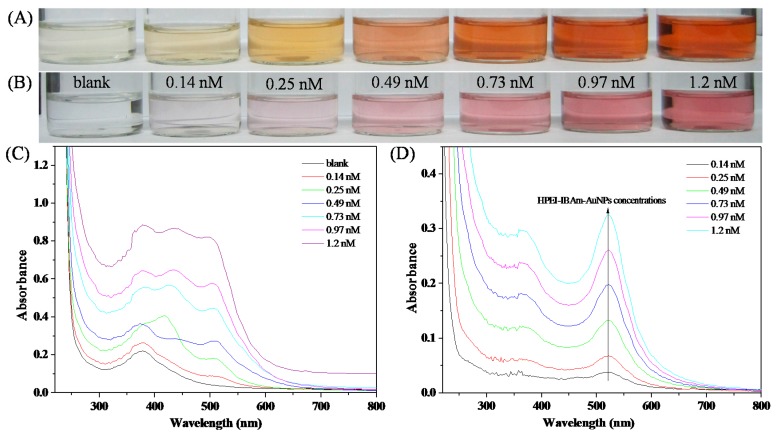
Photographs of HPEI-IBAm-AuNPs with different concentrations in the presence (**A**) and absence (**B**) of 0.573 mM Ag^+^; UV-vis absorption spectra of HPEI-IBAm-AuNP solution with different concentrations in the presence (**C**) and absence (**D**) of 0.573 mM Ag^+^.

As is well known, the sensitivity of AuNPs based colorimetric sensor is highly dependent on the concentration of AuNPs [7,9,10]. Therefore, the effect of the dosage of HPEI-IBAm-AuNPs on the response of the sensor was examined. According to the sensing mechanism mentioned above, we can know that Ag^0^ was directly deposited onto the surface of AuNPs, forming the Au-Ag core-shell NPs with a red to brown color change. Obviously, the higher concentration of AuNPs, the more Ag^0^ is needed to cover the AuNPs and to cause the color change, making the sensor had low sensitivity. In the presence of Ag^+^ at a given concentration, the lower concentration of AuNPs, the more Ag^0^ will be deposited on the surface of each single AuNP, which is more conducive to cause the color change and account for the lower detection limit (i.e., the higher sensitivity) but result in narrow linear range. Moreover, it can be seen from Figure 8 that the red of the sensor itself is not obvious at the concentration of AuNPs lower than 0.49 nM. When the concentration of AuNPs is higher than 0.49 nM, there are obvious differences in color (Figure 8A,B) and absorption spectra (Figure 8C,D) before and after the Ag^+^ detection, which is beneficial to colorimetric observation. Hence, taking into account both the sensitivity and the linear range of the colorimetric sensor, the concentration of 0.49 nM was chosen as the optimal dosage of AuNPs for Ag^+^ detection in our following experiments.

**Figure 9:**
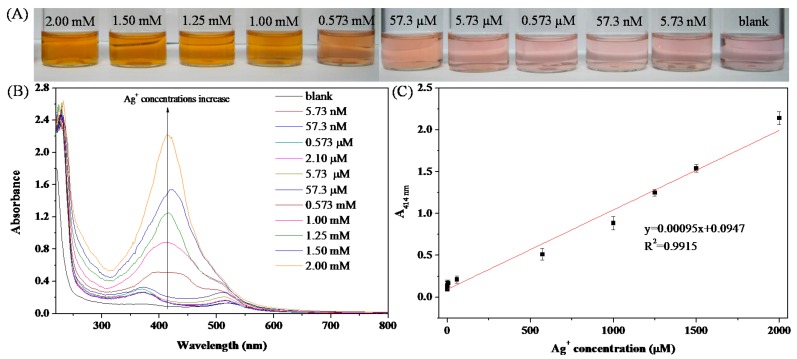
(**A**) Photographs and (**B**) UV-vis absorption spectra of 0.49 nM HPEI-IBAm-AuNPs solution in the presence of different Ag^+^ concentration (0–2.0 mM); (**C**) linear response (*A*_414 nm_ value of the absorbance at 414 nm) of the colorimetric assay against the Ag^+^ concentration range of 0–2.0 mM.

The detection limit and detection range are the key factors that affect the practicability of colorimetric sensor. Figure 9 shows the responses of the color change and UV-vis absorption spectra of 0.49 nM HPEI-IBAm-AuNPs solution with different concentrations of Ag^+^ (0–2 mM). Clearly, the color of HPEI-IBAm-AuNPs solution gradually changed from red through pale-brown to brown as the Ag^+^ concentration increased (Figure 9A), enabling 57.3 µM of Ag^+^ can be detected by the naked eye alone. With the increase of Ag^+^ concentration, the ~520 nm plasmon peak of HPEI-IBAm-AuNPs gradually blue shifted to 380–450 nm (Figure 9B), which depends on the thickness of Ag shell [10,32]. Here, we can see that the maximum absorption is centered at about 414 nm from Figure 9B. Therefore, the sensor responses were expressed by the absorption value at 414 nm (that is *A*_414 nm_). A good linear relationship was obtained between *A*_414 nm_ and Ag^+^ concentration in the range of 0–2.0 mM, with a correlation coefficient of *R*^2^ = 0.9915 (Figure 9C). Especially, there is still a good linear relationship when the concentration of Ag^+^ is as low as 5.73 nM to 5.73 µM with *R*^2^ = 0.9957 (Figure S10). The limit of detection (LOD) for Ag^+^ was calculated to be 7.22 nM (3σ, which was far below the guideline value of 0.1 mg/L ≈ 920 nM in drinking water defined by the EPA [33]). To the best of our knowledge, this non-aggregation based colorimetric sensor is one of the first reports on Ag^+^ detection with the widest range of concentration and is one of the most sensitive methods for Ag^+^ detection, compared with many other AuNPs-based sensors reported previously (Table S1). Therefore, this new colorimetric sensor based on the non-aggregation of AuNPs can be applied to the determination of Ag^+^ in different environments.

**Figure 10:**
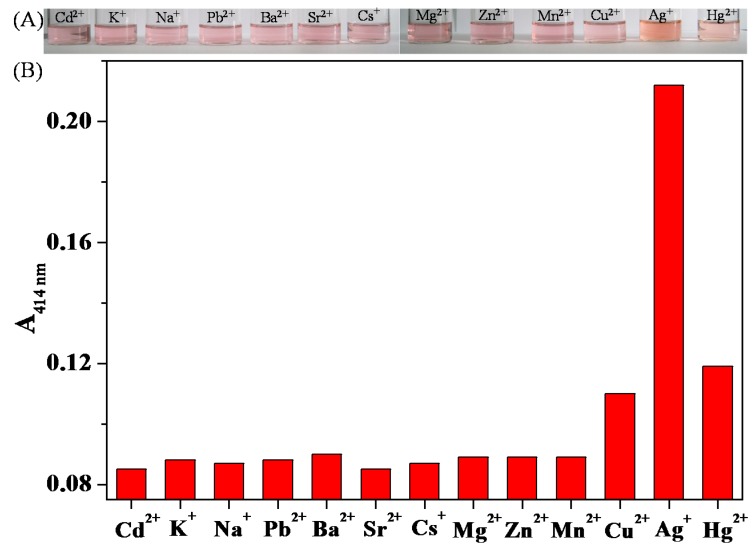
Selectivity of the sensor: (**A**) photographs and (**B**) response (*A*_414 nm_) of 0.49 nM HPEI-IBAm-AuNPs solution in the presence of various metal ions at the concentrations of 57.3 µM.

The selectivity of this colorimetric sensor for Ag^+^ was evaluated by testing the response of the sensor to other metallic ions including Cd^2+^, K^+^, Na^+^, Pb^2+^, Ba^2+^, Sr^2+^, Cs^+^, Mg^2+^, Zn^2+^, Mn^2+^, Cu^2+^, and Hg^2+^. As shown in Figure 10, only the HPEI-IBAm-AuNPs solution containing Ag^+^ ions caused obvious changes in the color (Figure 10A) and absorption spectra (Figure S11) because of the formation of Au-Ag core-shell NPs. The selectivity of this colorimetric sensor can be visualized with the naked eye as shown in Figure 10A. Meanwhile, it can be seen from the UV-Vis absorption spectra (Figure S10) that the *A*_414 nm_ value of this colorimetric sensor could be increased significantly to 0.212 upon the addition of Ag^+^, and most of the values for other metal ions were only approximately 0.09, except 0.11 of Cu^2+^ and 0.119 of Hg^2+^ (Figure 10B), indicating that the sensor showed great selectivity for Ag^+^ detection.

### 3.5. Ag^+^ Detection and Separation in Drinking Water

**Figure 11:**
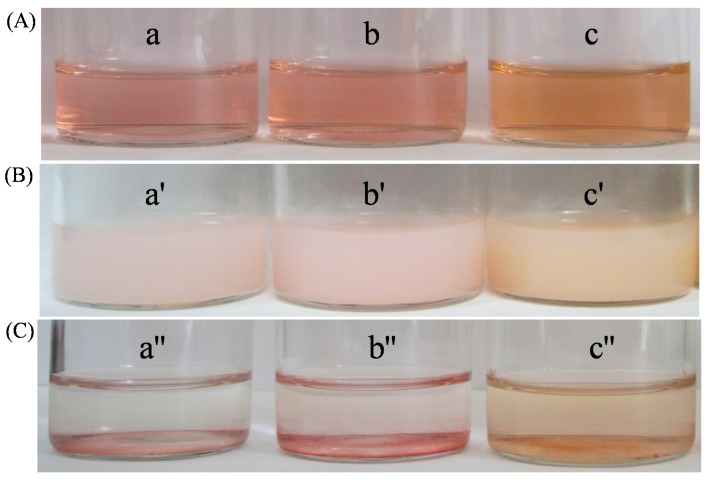

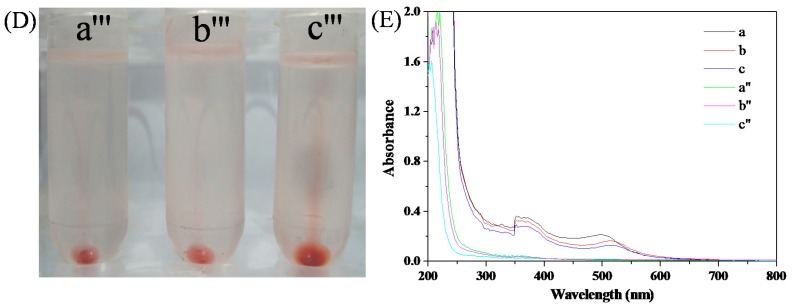
Colorimetric detection and separation of Ag^+^ in drinking water: (**A**) photographs of 0.49 nM HPEI-IBAm-AuNPs solution with (a) 60 µM, (b) 90 µM, and (c) 120 µM of Ag^+^; (**B**,**C**) represent the changes of 0.49 nM HPEI-IBAm-AuNPs solution with 60, 90, and 120 µM of Ag^+^ after keeping in a thermostatted water bath at 50 °C (10 degrees higher than CP) for 5 min and 6 h, respectively; (**D**) represents the change of 0.49 nM HPEI-IBAm-AuNPs solution with (a) 60 µM, (b) 90 µM, and (c) 120 µM of Ag^+^ after centrifugation at 50 °C; (**E**) absorption spectra of (**B**,**C**).

To further evaluate the applicability of the proposed colorimetric assay, the detection of Ag^+^ in drinking water was tested. According to the proposed colorimetric method, no changes in color and UV-Vis spectra were observed in the drinking water sample, indicating that Ag^+^ ions were not found. Then, the drinking water samples were spiked with 60, 90, and 120 µM of Ag^+^ ions (Table 1 and Figure 11A). The found concentrations based on the linear relationship between *A*_414 nm_ (Figure 11E) and Ag^+^ concentration were 62.1, 88.4, and 126.3 µM with the recoveries of 98.2%–105.3%, indicating that the proposed colorimetric assay can be used for the detection of Ag^+^ in real water samples and has high accuracy. The enrichment and separation of silver by the temperature sensitivity of the HPEI-IBAm were systematically studied. As shown in Figure 11B, the HPEI-IBAm-AuNPs solutions with 60, 90, and 120 µM of Ag^+^ became turbid when the ambient temperature was higher than the cloud point (CP) of the system. To achieve the full precipitation of HPEI-IBAm-AuNPs/Ag, the water bath temperature was set to 10 degrees higher than the respective CP of the silver-containing system. Additionally, the HPEI-IBAm-AuNPs/Ag composites can be separated by static stratification (Figure 11C) or centrifugation (Figure 11D). To determine the enriching efficiency of HPEI-IBAm-AuNPs toward Ag^+^, the concentration of residual Ag^+^ was determined by Inductively Coupled Plasma spectrometer (Table 1). The enriching efficiency of HPEI-IBAm-AuNPs toward Ag is very high (96.1%–97.3%), which is mainly due to the high affinity of AuNPs and amine groups of HPEI-IBAm-AuNPs to silver. All these results suggest that the proposed method based on the colorimetric response and thermoresponsive property of the HPEI-IBAm-AuNPs may be one of the most promising approaches for detecting, enriching, and separating silver (Scheme 1).

## 4. Conclusions

In summary, an in-situ reaction strategy for the preparation of stable AuNPs has been successfully achieved via a thermal process by using thermoresponsive HPEI-IBAm as templates, reducing agents, and stabilizers without the additional step of introducing a toxic small-molecule reductant. The HPEI-IBAm functionalized AuNPs exhibit excellent thermal stability up to 200 °C, high tolerance of a wide range of pH values (3–13), and high salt resistance. Thermoresponsive HPEI-IBAm not only serves as a green reductant and excellent stabilizer to prepare AuNPs via in-situ formation, but also serves as a trigger agent to cause an obvious color change of HPEI-IBAm-AuNPs solution in the presence of Ag^+^, which can be used for the colorimetric detection of Ag^+^. This new non-aggregation-based colorimetric sensor with a unique red-to-brown color change showed high stability (maintaining the color lasting without fading) and high selectivity. The colorimetric sensor showed high sensitivity with an extremely low detection limit of 7.22 nM and a good linear relationship in a wide concentration range of 0–2.0 mM (*R*^2^ = 0.9915), which is one of the most sensitive methods for Ag^+^ detection and is one of the first reports on Ag^+^ detection with the widest range of concentration. Even at low Ag^+^ concentrations (5.73 nM-5.73 µM), the sensing response still demonstrated high linearity (*R*^2^ = 0.9957). Moreover, this colorimetric sensor was successfully applied to detect Ag^+^ in drinking water. After the sensing detection, the AuNPs/Ag composites can be separated by heating the system above the cloud point of the polymer solution and then by performing static stratification or centrifugation, which can avoid secondary pollutions. Therefore, the proposed colorimetric sensor can not only achieve the detection of Ag^+^ with high sensitivity and high selectivity, but also can effectively separate and remove Ag^+^, thereby preventing and controlling its pollution to the environment. The HPEI-IBAm functionalized AuNPs can truly realize the integration of green preparation, detection, and separation, which serves the aims of energy saving, environmental protection, and sustainable development, and provides a basis to further develop multifunctional nano-materials.

## Figures and Tables

**Table 1:** Results of the Ag^+^ recovery and separation experiments performed in drinking water samples.

Added (µM)	Found (µM)	Recovery (%)	Residual Ag (µM)	Enriching efficiency (%)
60	62.1	103.5	1.9	96.8
90	88.4	98.2	3.5	96.1
120	126.3	105.3	3.2	97.3

## Supplementary Materials

The following are available online at www.mdpi.com/2073-4360/10/1/42/s1, Figure S1: ^1^H NMR spectra of HPEI-IBAm (A) and HPEI (B), Figure S2: comparison of FTIR spectra of HPEI-IBAm (A) and HPEI (B), Figure S3: the UV-Vis spectra of the HPEI-IBAm functionalized AuNPs after every evaporation at 110 °C and re-dispersion in Milli-Q water, Figure S4: (A) photographs of (a) the HPEI-IBAm functionalized AuNPs and (b) citrate-capped AuNPs after every evaporation at 110 °C and re-dispersion in Milli-Q water; (B) the maximum absorption peaks of the HPEI-IBAm functionalized AuNPs after each cycle’s re-dispersion; (C) the UV-Vis spectra of the citrate-capped AuNPs after each cycle’s re-dispersion, Figure S5: the UV-Vis spectra of the HPEI-IBAm functionalized AuNPs after every evaporation at 130 °C and re-dispersion in Milli-Q water, Figure S6: (A) photographs of (a) the HPEI-IBAm functionalized AuNPs and (b) citrate-capped AuNPs after every evaporation at 200 °C and re-dispersion in Milli-Q water; (B) the UV-Vis spectra of the HPEI-IBAm functionalized AuNPs after each cycle’s re-dispersion; (C) the UV-Vis spectra of the citrate-capped AuNPs after each cycle’s re-dispersion, Figure S7: the UV-Vis spectra of the HPEI-IBAm functionalized AuNPs under different salt concentrations, Figure S8: the UV-Vis spectra of the citrate-capped AuNPs after 24 h under different salt concentrations, Figure S9: (A) photographs and (B) UV-Vis spectra of (a) 1.2 nM citrate-capped AuNPs after (b) 1 h and (c) 24 h upon the addition of Ag^+^, Figure S10: Linear response (A414 nm value of the absorbance at 414 nm) of the colorimetric assay against the Ag+ concentration range of 5.73 nM to 5.73 μM. Figure S11: UV-Vis spectra of 0.49 nM HPEI-IBAm-AuNPs solution in the presence of various metal ions at the concentrations of 57.3 µM, Table S1: colorimetric sensors for Ag^+^ detection.
