# Supplementary material for: Green and Facile Synthesis of Highly Stable Gold Nanoparticles via Hyperbranched Polymer In-Situ Reduction and Their Application in Ag+ Detection and Separation
Source: Polymers (Basel). 2018 Jan 3;10(1):42. doi: 10.3390/polym10010042 (PMC6415124; doi:10.3390/polym10010042)
Supplement: Supplementary file 1 [file polymers-10-00042-s001.pdf]

Green and Facile Synthesis of Highly Stable Gold Nanoparticles via Hyperbranched Polymer In-situ Reduction and Their Application in Ag^+ Detection and Separation

Xunyong Liu, Chenxue Zhu, Li Xu, Yuqing Dai, Yanli Liu and Yi Liu

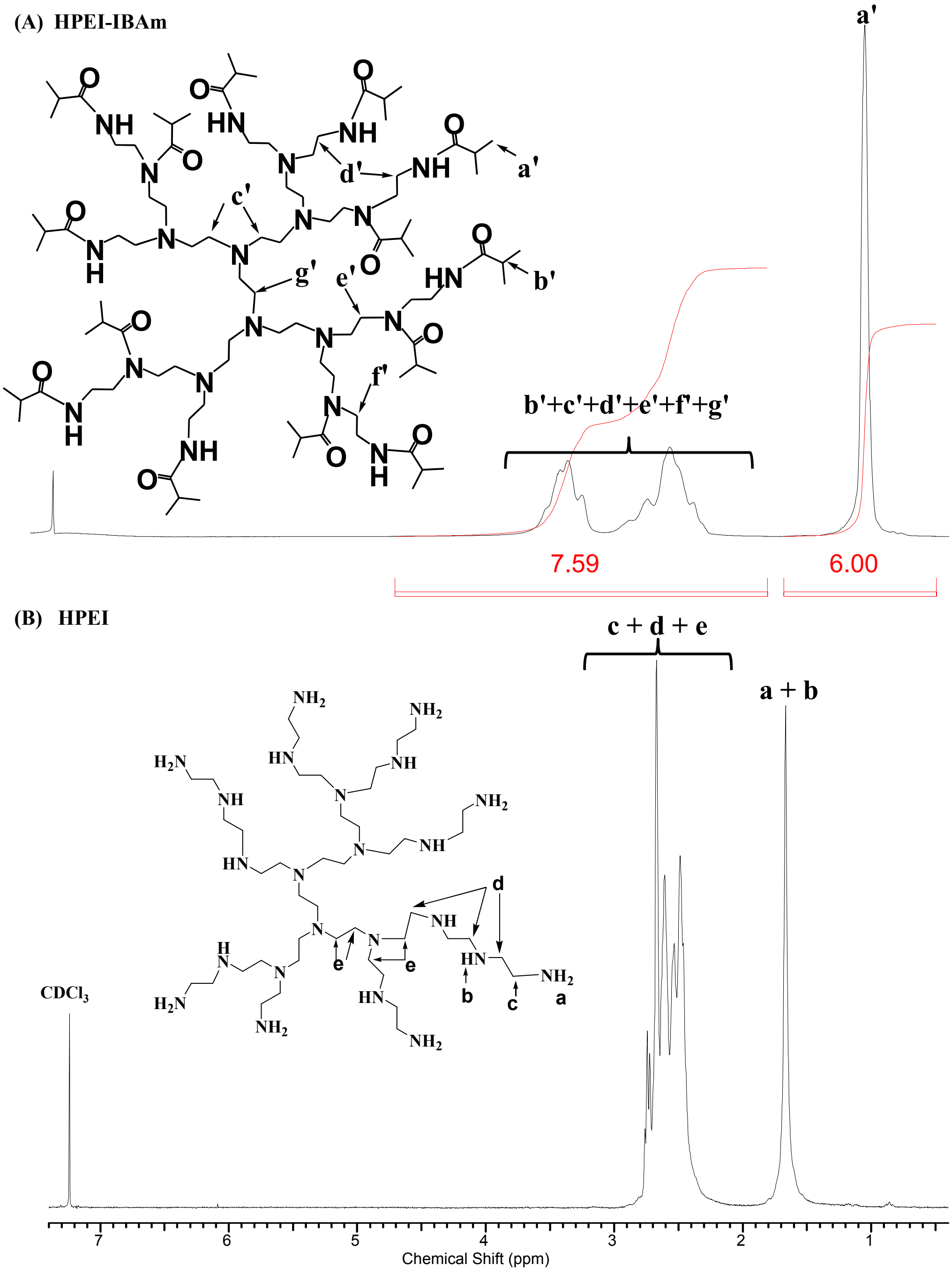

Figure S1. 1H NMR spectra of HPEI-IBAm (A) and HPEI (B).

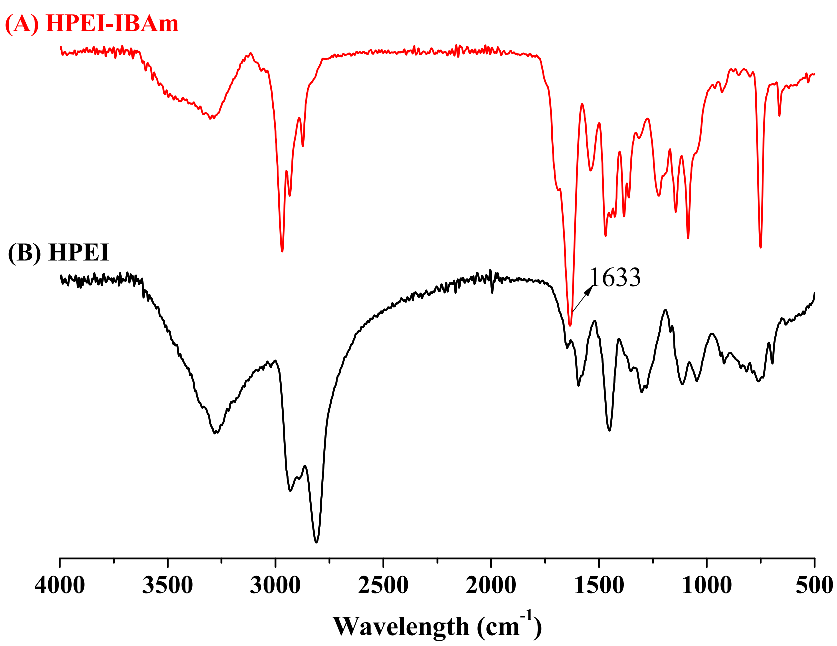

Figure S2. Comparison of FTIR spectra of HPEI-IBAm (A) and HPEI (B).

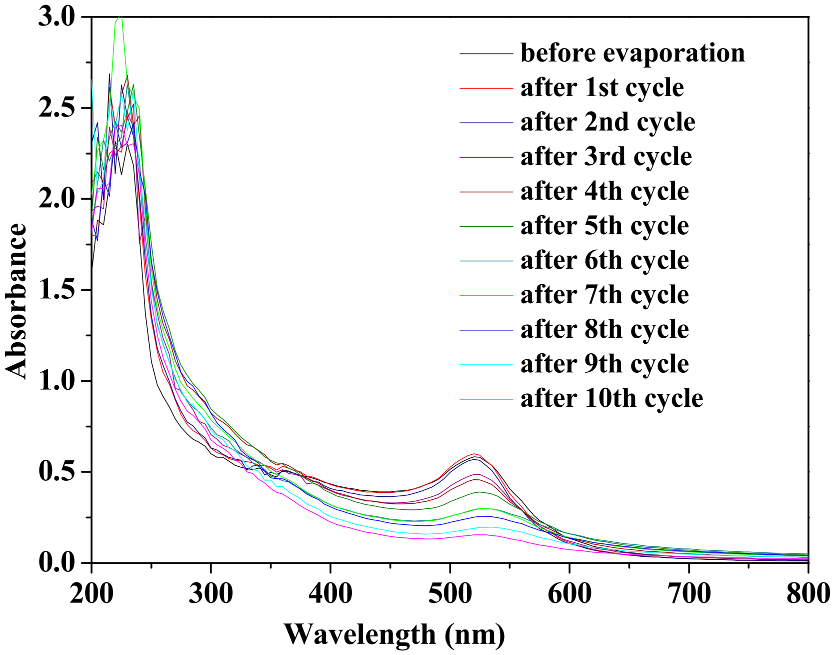

Figure S3. The UV-Vis spectra of the HPEI-IBAm functionalized AuNPs after every evaporation at 110 °C and re-dispersion in Milli-Q water.

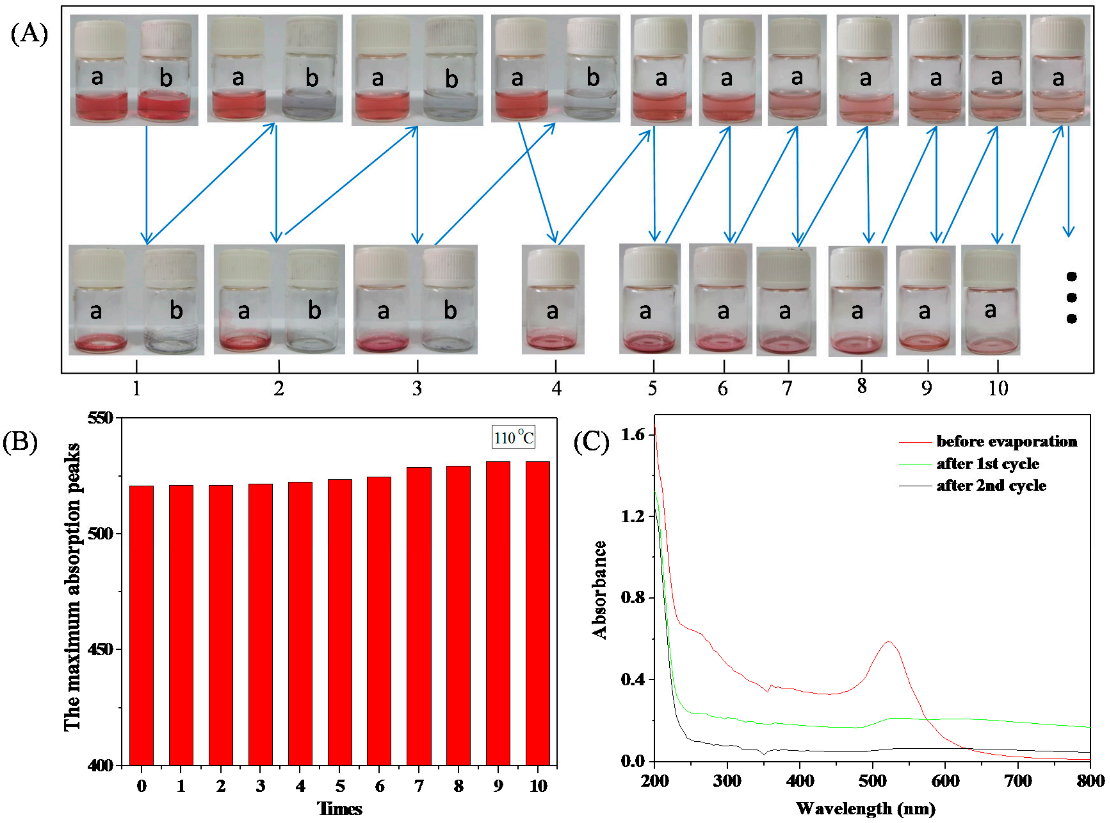

Figure S4. (A) Photographs of (a) the HPEI-IBAm functionalized AuNPs and (b) citrate-capped AuNPs after every evaporation at 110 °C and re-dispersion in Milli-Q water; (B) The maximum absorption peaks of the HPEI-IBAm functionalized AuNPs after each cycle's re-dispersion; (C) The UV-Vis spectra of the citrate-capped AuNPs after each cycle's re-dispersion.

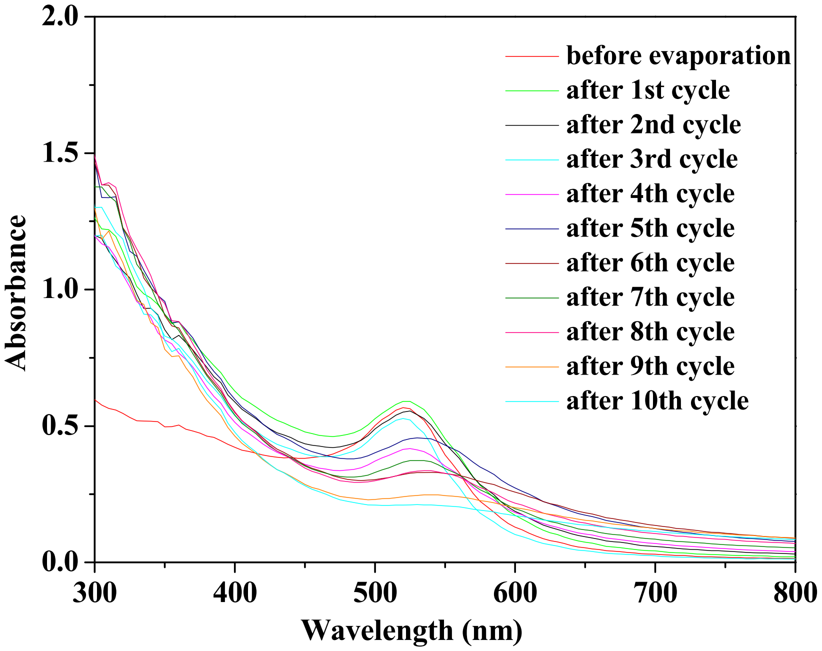

Figure S5. The UV-Vis spectra of the HPEI-IBAm functionalized AuNPs after every evaporation at

130 °C and re-dispersion in Milli-Q water.

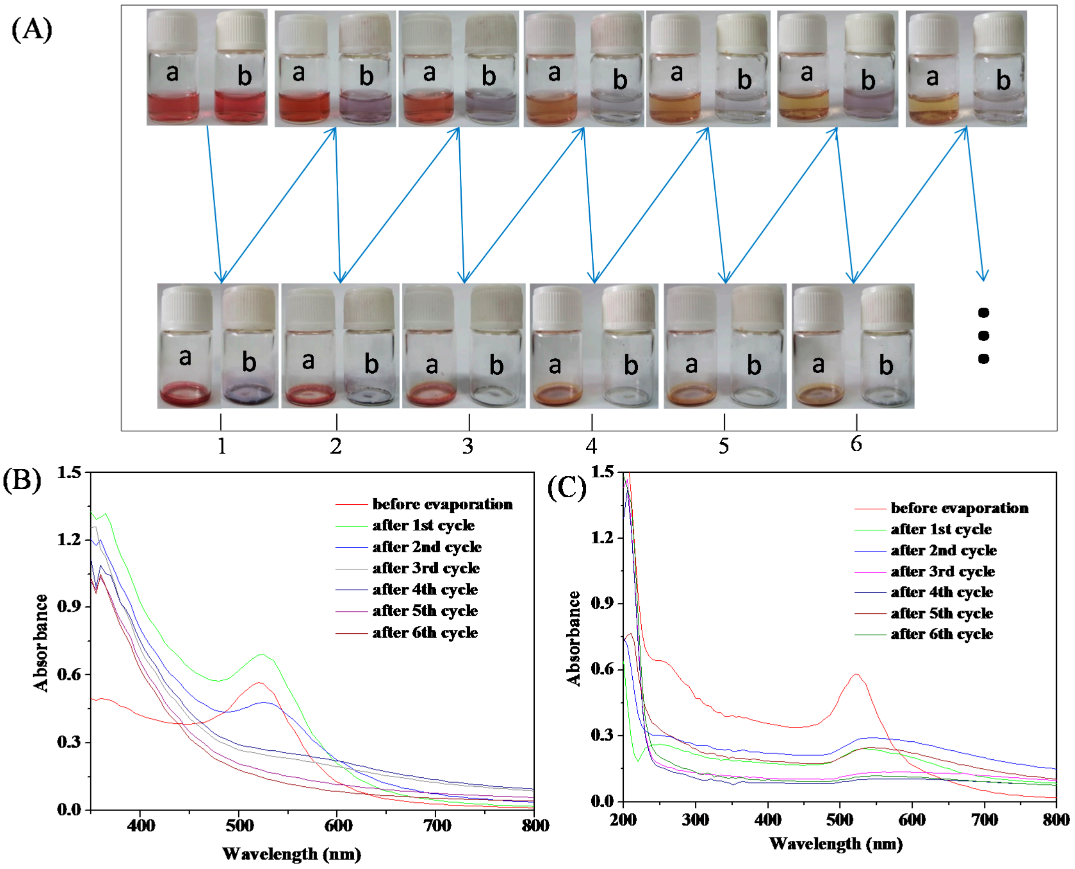

Figure S6. (A) Photographs of (a) the HPEI-IBAm functionalized AuNPs and (b) citrate-capped AuNPs after every evaporation at 200 °C and re-dispersion in Milli-Q water; (B) The UV-Vis spectra of the HPEI-IBAm functionalized AuNPs after each cycle's re-dispersion; (C) The UV-Vis spectra of the citrate-capped AuNPs after each cycle's re-dispersion.

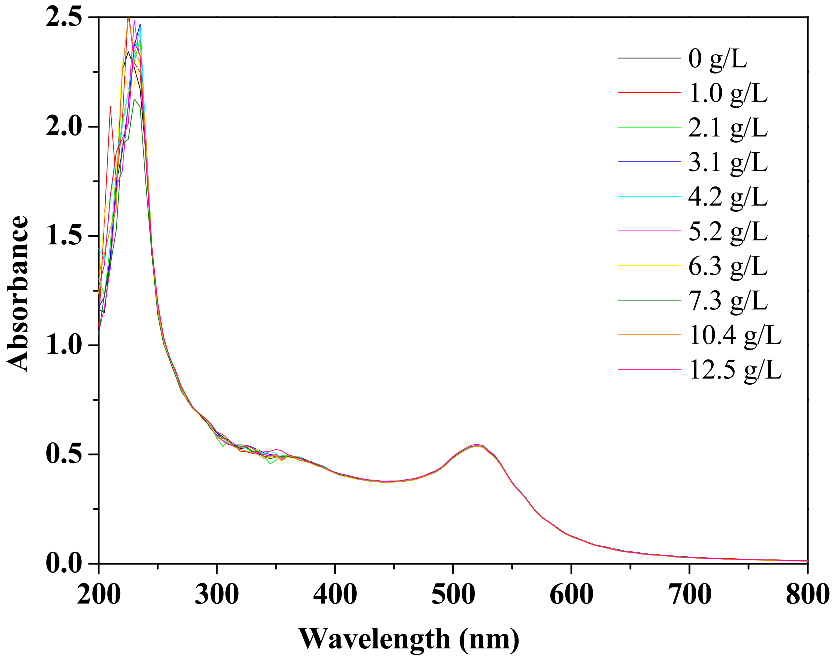

Figure S7. The UV-Vis spectra of the HPEI-IBAm functionalized AuNPs under different salt concentrations.

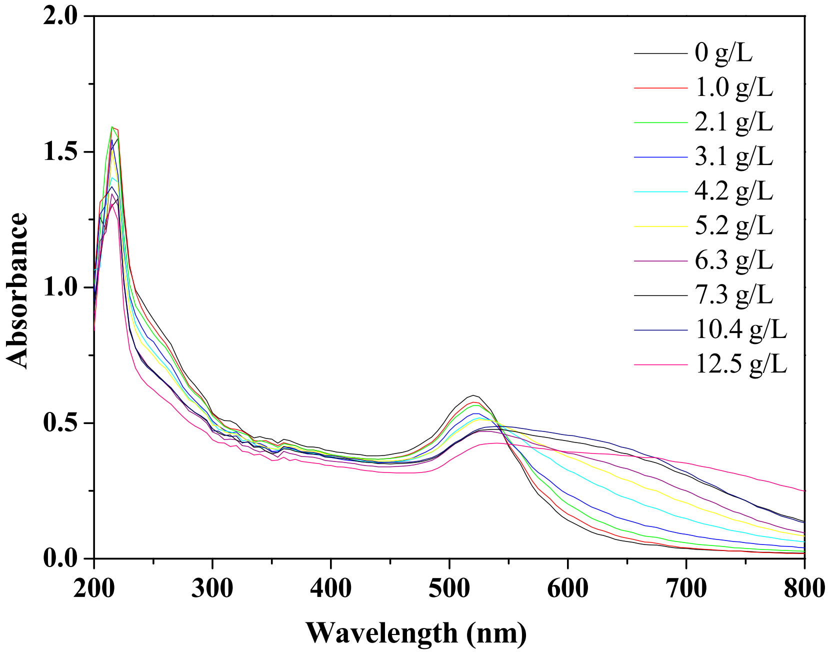

Figure S8. The UV-Vis spectra of the citrate-capped AuNPs after 24 h under different salt concentrations.

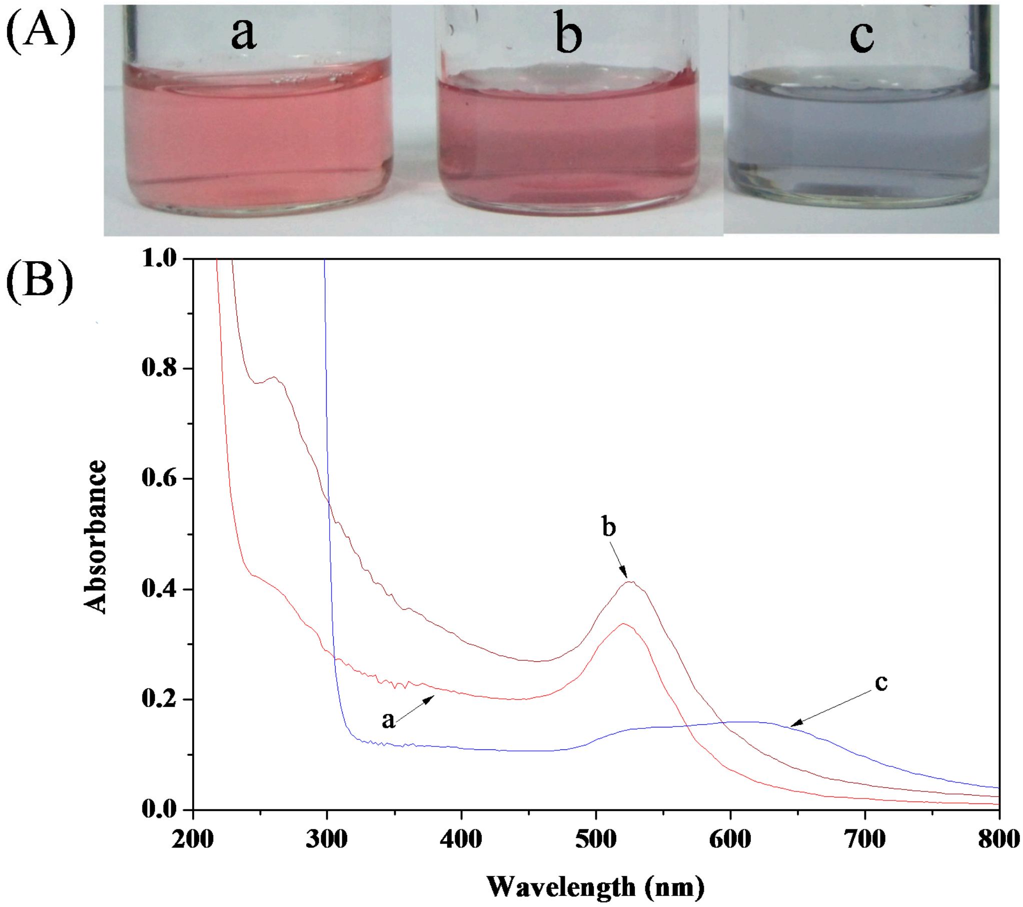

Figure S9. (A) Photographs and (B) UV-Vis spectra of (a) 1.2 nM citrate-capped AuNPs after (b) 1 h and (c) 24 h upon the addition of Ag^+.

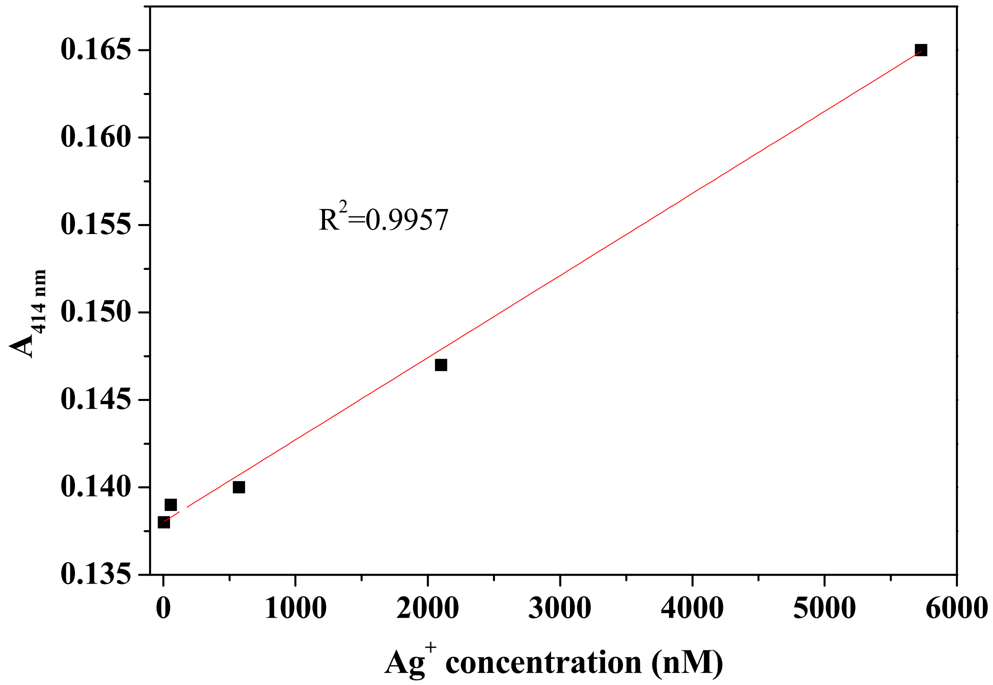

Figure S10. Linear response (A414 nm value of the absorbance at 414 nm) of the colorimetric assay against the Ag+ concentration range of 5.73 nM to 5.73 M.

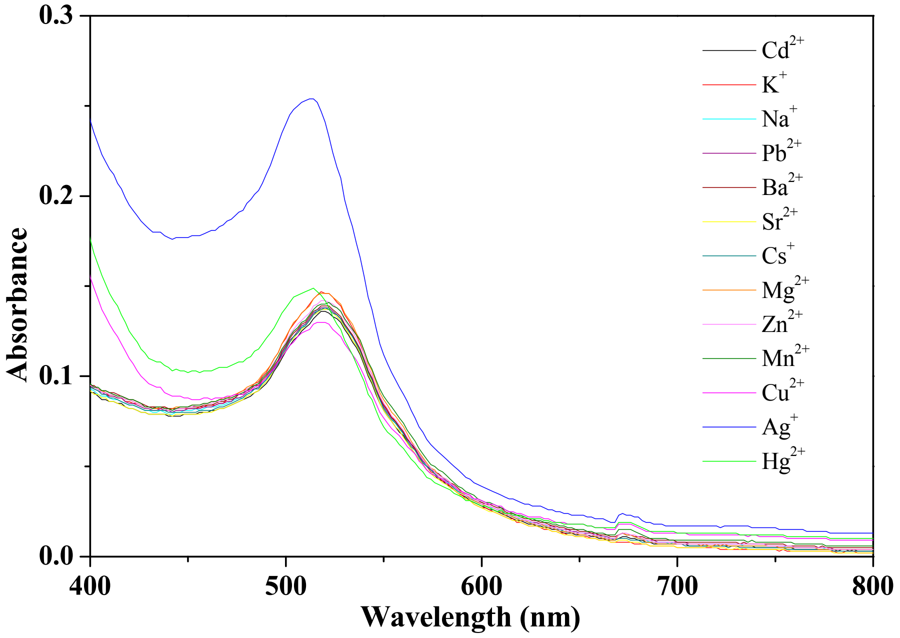

Figure S11. UV-Vis spectra of 0.49 nM HPEI-IBAm-AuNPs solution in the presence of various metal ions at the concentrations of 57.3 M.

Table S1. Colorimetric sensors for Ag+ detection.

Probe	Detection techniques	LOD (nM)	Detection range	Ref
Tween 20-AuNPs	Colorimetry and absorption	100	400-1000 nM	[1]
Pyridines-functionalized AuNPs	Colorimetry and absorption	1000	—[a]	[2]
Oligonucleotide/AuNPs	Colorimetry	62	0.13-1.12 μM	[3]
Tween 20-AuNPs	Colorimetry and absorption	10	1-8 μM	[4]
Core-shell AuNPs	Absorption	10	10 nM-0.1 mM	[5]
Gold nanorod (GNR)	Absorption	10	10 nM-1 mM	[6]
Cationic polymer-directed AuNPs	Colorimetry and absorption	48.6	100-1000 nM	[7]
DNA probe and AuNPs	Colorimetry and absorption	500	1.5-4 μM	[8]
Multidentate ligand-AuNPs	Colorimetry and absorption	8.76	8.76 nM-0.13 mM	[9]
Thermoresponsive hyperbranched polymer-AuNPs	Colorimetry and absorption	7.22	0-2.0 mM	This work

[a]The detection range was not given.

References

[1] C. Y. Lin, C. J. Yu, Y. H. Lin, W. L. Tseng, Colorimetric Sensing of Silver(I) and Mercury(II) Ions Based on an Assembly of Tween 20-Stabilized Gold Nanoparticles. Anal. Chem. 82 (2010) 6830-6837.

[2] A. Alizadeh, M. M. Khodaei, Z. Hamidi, M. Bin Shamsuddin, Naked-eye colorimetric detection of Cu^{2+} and Ag^{+} ions based on close-packed aggregation of pyridines-functionalized gold nanoparticles. Sens. Actuator B-Chem. 190 (2014) 782-791.

[3] C. K. Wu, C. Xiong, L. J. Wang, C. C. Lan, L. S. Ling, Sensitive and selective localized surface plasmon resonance light-scattering sensor for Ag+ with unmodified gold nanoparticles. Analyst 135 (2010) 2682-2687.

[4] T. T. Lou, Z. P. Chen, Y. Q. Wang, L. X. Chen, Blue-to-Red Colorimetric Sensing Strategy for Hg^{2+} and Ag^{+} via Redox-Regulated Surface Chemistry of Gold Nanoparticles. ACS Appl. Mater. Interfaces 3 (2011) 1568-1573.

[5] H. Huang, C. Qu, X. Liu, S. Huang, Z. Xu, B. Liao, Y. Zeng, P. K. Chu, Preparation of Controllable Core-Shell Gold Nanoparticles and Its Application in Detection of Silver Ions. ACS Appl. Mater. Interfaces 3 (2011) 183-190.

[6] H. Huang, S. Chen, F. Liu, Q. Zhao, B. Liao, S. Yi, Y. Zeng, Multiplex Plasmonic Sensor for Detection of Different Metal Ions Based on a Single Type of Gold Nanorod. Anal. Chem. 85 (2013) 2312-2319.

[7] F. Z. Wang, Y. G. Wu, S. S. Zhan, L. He, W. T. Zhi, X. X. Zhou, P. Zhou, A Simple and Sensitive Colorimetric Detection of Silver Ions Based on Cationic Polymer-Directed AuNPs Aggregation. Aust. J. Chem. 66 (2013) 113-118.

[8] Z. H. Qing, X. X. He, K. M. Wang, Z. Zou, X. Yang, J. Huang, G. P. Yan, Colorimetric multiplexed analysis of mercury and silver ions by using a unimolecular DNA probe and unmodified gold nanoparticles. Anal. Methods 4 (2012) 3320-3325.

[9] Y. Liu, J. Dai, L. Xu, X. Liu, J. Liu, G. Li, Red to brown to green colorimetric detection of Ag^{+} based on the formation of Au-Ag core-shell NPs stabilized by a multi-sulfhydryl functionalized hyperbranched polymer. Sens. Actuators, B 237 (2016) 216-223.
